# Appendicular peritonitis in *situs inversus totalis*: a case report

**DOI:** 10.1186/1752-1947-4-134

**Published:** 2010-05-11

**Authors:** Mamadou Cissé, Alpha O Touré, Ibrahima Konaté, Madieng Dieng, Ousmane Ka, Fodé B Touré, Abdarahmane Dia, Cheikh T Touré

**Affiliations:** 1 Clinique Chirurgicale, Hôpital Aristide Le Dantec, Dakar, Avenue Pasteur, BP 3001, Sénégal

## Abstract

**Introduction:**

*Situs inversus *is a congenital anomaly characterized by the transposition of the abdominal viscera. When associated with dextrocardia, it is known as *situs inversus totalis*. This condition is rare and can be a diagnostic problem when associated with appendicular peritonitis.

**Case presentation:**

We report the case of a 20-year-old African man who presented to the emergency department with a 4-day history of diffuse abdominal pain, which began in his left iliac region and hypogastrium. After examination, we initiated a surgical exploration for peritonitis. We discovered a *situs inversus *at the left side of his liver, and his appendix was perforated in its middle third. A complementary post-operative thoracic and abdominal tomodensitometry revealed a *situs inversus totalis*.

**Conclusion:**

Appendicular peritonitis in *situs inversus *is a rare association that can present a diagnostic problem. Morphologic exploration methods such as ultrasonography, tomodensitometry, magnetic resonance imaging, and laparoscopy may contribute to the early management of the disease and give guidance in choosing the most appropriate treatment for patients.

## Introduction

*Situs inversus *is a congenital anomaly characterized by the transposition of the abdominal viscera. It may or may not be associated with dextrocardia, also known as *situs inversus totalis *[1,2]. Generally, this rare genetic anomaly is discovered incidentally, often when a radiographic assessment of a patient is undertaken, particularly to investigate an abdominal infection. We report a case of *situs inversus *discovered in relation to the treatment of generalized acute peritonitis of appendicular origin. This case is particularly interesting because of the scarcity of this association and the diagnostic difficulties that may arise because of unusual symptoms.

## Case presentation

A 20-year-old African man presented to the emergency department at the Aristide Le Dantec hospital with 4-day history of diffuse abdominal pain in his left iliac region and hypogastrium. This pain was associated with bilious vomiting and fever. On examination, he was found to be in a good general condition. He had a fever at 40°C, a pulse rate of 120/minute, and blood pressure of 120/70 mm Hg. His physical examination revealed a generalized abdominal tenderness predominantly over his left lower and hypogastric quadrants.

**Figure 1:**
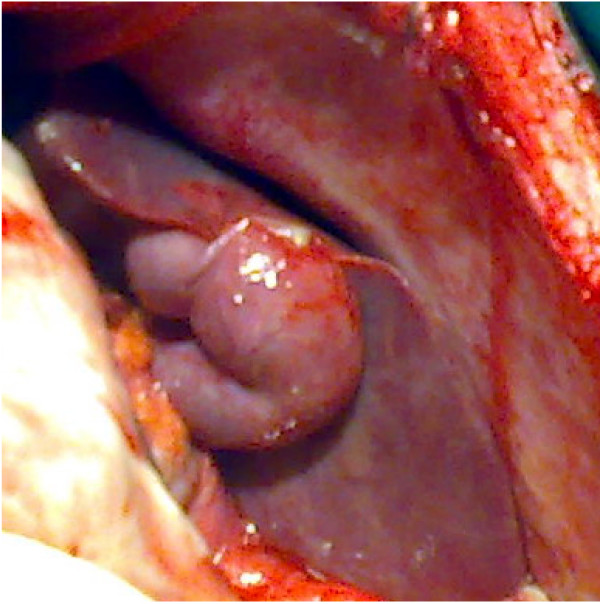
**Peri-operative view of *situs inversus *with left-sided liver and gallbladder**.

**Figure 2:**
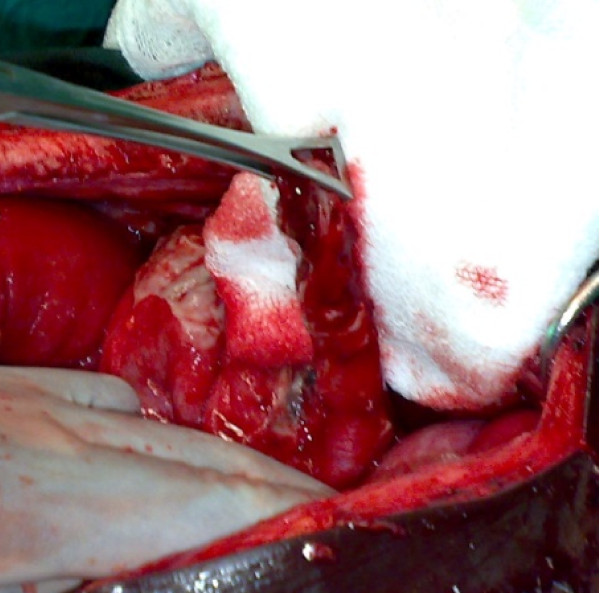
**Perforated appendix in the left iliac fossa**.

Laboratory investigations showed that he had a white blood cell count of 18,900/mm^3 ^with 93% neutrophils, 42% hematocrit, and platelets at 323,000/mm^3^. An X-ray of our patient's abdomen showed small bowel loops and a diffuse grayness. After a pre-operative reanimation, a median laparotomy was performed. The exploration showed an acute generalized peritonitis with 300 mm^3 ^of pus, false membranes, *situs inversus *(Figure 1), and a phlegmonous pelvic appendix perforated in its middle third (Figure 2). An appendectomy and peritoneal toilet were subsequently performed.

**Figure 3:**
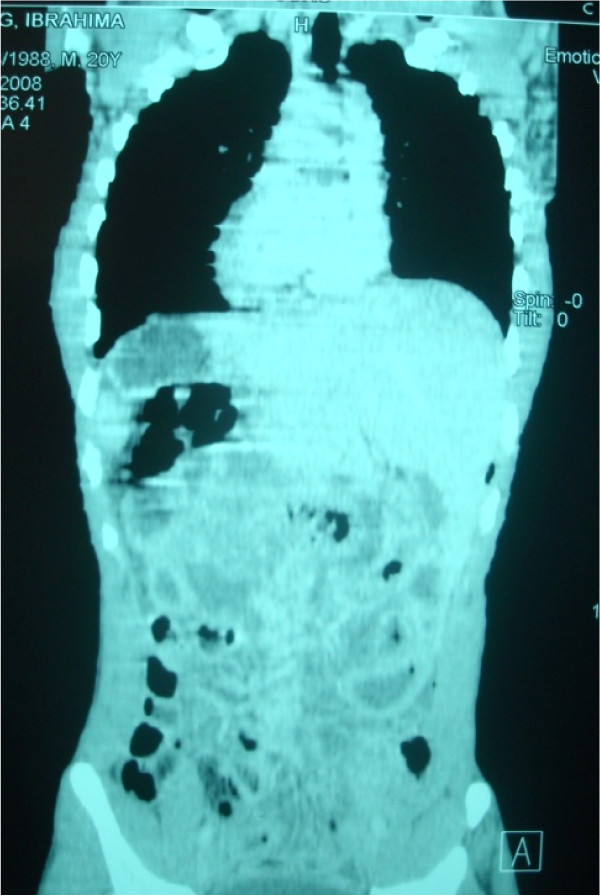
**Frontal scan of the dextrocardia and the left-sided liver shadow**.

**Figure 4:**
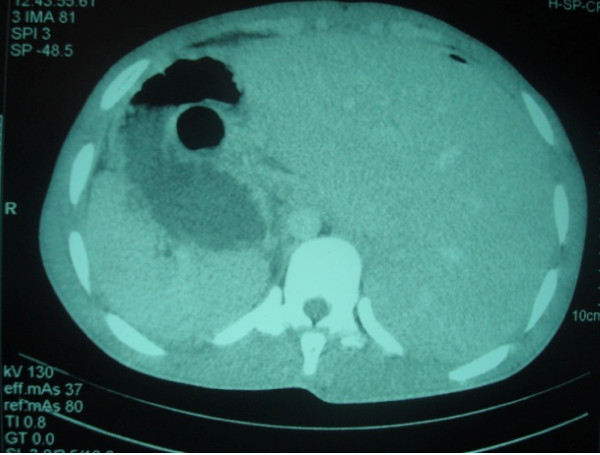
**Left-sided liver and right-sided spleen**.

A post-operative abdominal tomodensitometry with a frontal view of our patient's abdomen and lower chest was performed to assess his condition. This revealed a *situs inversus totalis *with dextrocardia and a left-sided liver (Figures 3 and 4). A bacteriologic analysis of the pus isolated *Bacteroides fragilis *sensitive to the combination of amoxicillin and clavulanic acid. Surgical pathology confirmed acute appendicitis with suppurative necrosis of his serous membrane. No post-operative complication was noted, and he was discharged home eight days after his operation.

## Discussion

*Situs inversus *is a positional anomaly that rotates the abdominal internal viscera. It is known as *situs inversus totalis *when it is associated with a transposition of the thoracic organs. *Situs inversus *is a rare congenital anomaly with an incidence in the population of only 0.001% to 0.01% [1,2] with a male-to-female ratio of 3:2 [3]. Its transmission mode is autosomal recessive, but its precise genetic mechanism has yet to be identified [1,3].

*Situs inversus *results from a rotation in the opposite direction of the viscera during the development of the embryo [2,3]. Patients with *situs inversus *may face diagnostic problems because of the unusual localizations of their symptoms. In the case of our patient's pain in the left iliac fossa, the differential diagnosis we made was extensive. Even in patients without *situs inversus*, the right iliac appendicular symptoms would be found in only 60% of cases [1,3]. The presence of symptoms in the left iliac fossa in the absence of *situs inversus *may be due to an abnormally long appendix projected to the left, or to intestinal hyperkinesis.

A study of 71,000 patients appendicular symptoms found that 0.04% of cases involved left iliac localization, comprising 0.024% with abdominal *situs inversus *and 0.016% with *situs inversus totalis *[3,4]. Until 2008, fewer than 10 cases of appendicitis associated with *situs inversus *were reported in the literature [3]. Half of these patients reported pain in their right iliac fossa despite the presence of *situs inversus *[1]. Therefore, given the scarcity of this association, the diagnosis of appendicitis with *situs inversus *is not automatically evoked, which delays the appropriate management of patients. As a consequence, as in the case of our patient, peritoneal diffusion may eventually develop.

Meanwhile, the usual differential diagnosis of left lower quadrant abdominal pain in an adult man includes, among others, sigmoid diverticulitis, epididymitis, bowel obstruction, psoas abscess, and, in this rare instance, *situs inversus *with acute appendicitis. Medical imaging can help clinicians to arrive at a correct diagnosis. Abdominal X-ray, ultrasonography, and tomodensitometry can also facilitate an accurate and early diagnosis if a patient is unaware of this positional anomaly [1,3,4]. Medical imaging can also guide the appropriate therapeutic choice, surgical indication, and type and location of the incision [4]. The contribution of laparoscopy is undeniably useful in these situations, as it favors a minimally invasive surgical approach in diagnostics and treatment [5].

## Conclusion

The occurrence of appendicitis with *situs inversus *is very rare. Very few cases have been reported in the literature. This condition poses a diagnostic problem that can be decreased by including morphologic exploration methods such as ultrasonography, tomodensitometry, and laparoscopy. These procedures allow the early management of the disease and guide therapeutic choices.

## Competing interests

The authors declare that they have no competing interests.

## Authors' contributions

MC and AOT performed the surgical procedure and drafted the case report. IK and OK interpreted and analyzed the tomodensitometry findings. MD participated in the diagnostic and therapeutic decisions. AD and CTT made major contributions to writing the manuscript. All authors read and approved the final manuscript.

## Consent

Written informed consent was obtained from our patient for publication of this case report and any accompanying images. A copy of the written consent is available for review by the Editor-in-Chief of this journal.
